# Assessment of cells in the ascitic fluid of women with ovarian hyperstimulation syndrome: the clinical implications for subsequent ovarian malignancy

**DOI:** 10.1186/1477-7827-11-91

**Published:** 2013-09-12

**Authors:** Ioannis Hatzipetros, Peter M Gocze, Katalin Cziraky, Kalman Kovacs, Endre Kalman, Balint Farkas

**Affiliations:** 1 Department of Obstetrics and Gynaecology, University of Pecs, Clinical Centre, Edesanyak Str. 17, 7624 Pecs, Hungary; 2 Department of Pathology, University of Pecs, Pecs, Hungary

## Abstract

**Background:**

Although some studies have reported a potential connection between ovulation induction therapy (OIT) and malignant ovarian diseases, the results have been inconclusive. In the present study, we sought to determine whether women undergoing OIT at our *in vitro* fertilization (IVF) clinic, especially those with severe ovarian hyperstimulation syndrome (OHSS) and suspicious cytologic findings, were at risk for developing malignant ovarian tumours after treatment.

**Methods:**

Patients who underwent OIT at our IVF clinic were enrolled in this study and assessed for any evidence of malignant ovarian tumours. Patients who developed severe OHSS as a result of OIT were treated with a culdocentesis. Cells from the ascitic fluid were cytologically scored for abnormality and malignancy. Peripheral blood samples were obtained from patients with severe OHSS to determine serum levels of the tumour markers (CA-125 and HE4) that were used to calculate the Risk for Ovarian Malignancy Algorithm (ROMA) index.

**Results:**

Follow-up data were available for 1,353 of the 1,587 patients (85%) who underwent OIT at our IVF clinic between January 2006 and December 2012. Twenty-three patients (1.4%) were hospitalized with OHSS. Culdocentesis was performed 16 times in nine patients with severe OHSS (age range, 23–34 years; mean, 27.1 years). Although cytological examination of the ascitic cells of these patients suggested malignant ovarian neoplasia, over the course of the observation period, the ovarian volume gradually decreased and became normal. Subsequent cytological and histological examinations failed to find evidence of any malignant tumours in these nine patients. None of the 1,353 participants who underwent OIT developed any malignant ovarian tumours during the study period. Moreover, none of the 462 patients who were in our ovarian tumour registry were also participants in the IVF program.

**Conclusions:**

The presence of atypical cells in the ascitic fluid of women with severe OHSS does not likely indicate malignancy; therefore, radical surgical intervention is not justified. The risk of malignancy is minimal shortly after OIT. At our centre, OIT has not been associated with any cases of ovarian tumour.

## Background

Ovarian hyperstimulation syndrome (OHSS) is an iatrogenic complication of ovarian induction therapy (OIT) that may be observed after stimulation with human chorionic gonadotropin or after the spontaneous luteinizing hormone peak. In OHSS, increased vascular permeability leads to subsequent fluid accumulation, especially in the abdominal cavity [1], with symptoms appearing 5 to 10 days after gonadotropin administration. The risk factors for OHSS include young age, polycystic ovarian syndrome, and a medical history of hyper-response to gonadotropins [2]. Early prediction of OHSS is crucial for prompt treatment. Diagnostic measures for predicting OHSS include an antral follicle count of ≥14 on a transvaginal ultrasound (82% sensitivity and 89% specificity) [3] and a basal anti-Müllerian hormone serum level of ≥ 3.5 ng/mL (90.5% sensitivity, 81.3% specificity) [4].

There are three forms of OHSS that can be distinguished by clinical signs and laboratory findings. Mild OHSS is a relatively common side effect of controlled ovarian stimulation that affects up to one-third of patients undergoing *in vitro* fertilization (IVF). Moderate and severe forms of OHSS have a combined incidence ranging from 3% to 8% [5]. The clinical consequences of mild and moderate OHSS are very minor. However, severe OHSS is a potentially life-threatening condition with symptoms that include ovarian enlargement, hydrothorax, hemoconcentration, salt and water dysregulation, oliguria, thromboembolic disease, and coagulation abnormalities. Approximately 1.4% of OHSS cases are severe, and severe OHSS is associated with a mortality risk of 1 in 450,000 to 500,000 [6].

Several studies have suggested a possible connection between OIT and ovarian tumours. For example, researchers have observed cases of struma ovarii [7], folliculoma [8], serous papillary carcinoma [9], mucinous cystadenoma [10], serous papillary cystadenoma [10], epithelial ovarian carcinoma [11], and cystadenocarcinoma [12] during and/or after OIT. In a case–control study performed in Israel between 1990 and 1993, Shushan *et al. *[13] concluded that OIT with human menopausal gonadotropin might increase the risk of epithelial ovarian malignancies, specifically borderline ovarian tumours. However, in some studies, hyperstimulation-induced reversible histological changes may have been grouped with malignant disease [14,15].

In light of the potential confounders present in previous data, further research is needed to clarify the relationship between OIT and ovarian malignancy. The aim of this study was to determine whether women undergoing OIT at our IVF clinic, especially those with severe OHSS and suspicious cytologic findings, were at risk for developing malignant ovarian tumours after treatment. Cells from the ascitic fluid recovered from patients with severe OHSS were characterized to determine if they were cytologically abnormal and whether the cytology indicated the presence of an ovarian malignancy.

## Methods

### Patients and study design

This prospective study was approved by the University of Pecs Institutional Ethical Review Board. Patients were included in this study if they were treated with OIT at the Clinical Centre of the University of Pecs Department of Obstetrics and Gynaecology/Reproductive Centre between January 2006 and December 2012 and provided their written informed consent to participate. Patients were questioned in person or surveyed by a mailed questionnaire about any current or past treatments for malignant ovarian tumours.

### Evaluation of the abdominal fluid

From January 2006 to December 2012, nine IVF clinic patients developed severe OHSS. Ovarian hyperstimulation was classified into three grades according to the severity of the symptoms, signs, and laboratory findings (Rizk and Aboulghar, 1999). These patients were treated with standard drug therapies, including a macrolide, intravenous fluids, clexane, and aspirin, as well as ultrasound-controlled culdocentesis [16]. During the culdocentesis, ascitic fluid was obtained from these patients for further analysis.

The ascitic fluid was placed in a centrifuge tube on ice and centrifuged at 400 × *g* for 10 minutes. Most of the fluid was decanted, and the resulting pellet was suspended with the remaining small amount of ascitic fluid using a shaker. A smear was made according to the usual procedures. The smear was fixed for 30 to 60 minutes in a 1/1 (v/v) mixture of ether and ethyl alcohol. After dehydration, the smears were stained with GIEMSA, evaluated by the Papanicolaou method [17] and analysed in the Clinical Cytological Laboratory. The samples were then analysed with visual light microscopy. The presence of abnormal cells was based on the assessment of the cellular shape (flat, sheet-like appearance and well-defined borders) and nuclear and nucleolar size differences in comparison to normal cells. The histological examination of the ovaries was carried out at the Department of Pathology of the Clinical Centre of the University of Pecs. The following cytologic grading system was used: P I, no abnormal or atypical cells; P II, atypical cells present, but with benign cytological appearance; P III, atypical cells suspicious for malignancy; P IV, cells diagnostic for malignancy; and P V, a large numbers of malignant cells.

### Measurement of serum levels of tumour markers

Peripheral blood samples were obtained from patients after the diagnosis of severe OHSS but before any interventions were performed. The serum concentrations of CA-125 (Fujirebio Diagnostics, Malvern, PA, USA; Catalogue # 400–10, Lot # 29192) and HE4 (Fujirebio Diagnostics; Catalogue # 404–10, Lot # 28374) were determined by a quantitative sandwich enzyme-linked immunosorbent assay (ELISA) according to the manufacturer’s protocol. Serum concentrations were calculated with the Optima 2.10 R2 built-in data calculator software.

### Risk for Ovarian Malignancy Algorithm (ROMA) index

The ROMA index is based on the serum levels of HE4 and CA-125 as measured by ELISA or calculated with an Excel spreadsheet using pre-set formulas to generate the predictive index (PI) for epithelioid ovarian cancer according to the following equation for premenopausal women: PI = −12 + 2.38 × ln [HE4] + 0.0626 × ln [CA125]. The ROMA value is calculated as follows: ROMA value (%) = exp (PI) / [1 + exp (PI)] × 100. According to the manufacturer’s manual (Fujirebio Diagnostic Inc., Malvern, PA, USA), a ROMA index equal to or greater than 13.1% is associated with a high risk of epithelioid ovarian cancer in premenopausal women [18].

## Results

Between January 2006 and December 2012, a total of 1,587 patients underwent OIT in 4,892 cycles at our IVF clinic. Of these patients, 23 (1.4%) were hospitalized with severe OHSS. We obtained follow-up data from 1,353 (85%) patients who underwent OIT and all 23 who developed OHSS. Of the 1,353 patients who underwent OIT at our clinic and were followed-up in this study, none developed a malignant ovarian tumour during the study period. A review of the local institutional registry revealed that none of the 462 registered patients with malignant ovarian tumours had participated in our IVF program.

**Figure 1:**
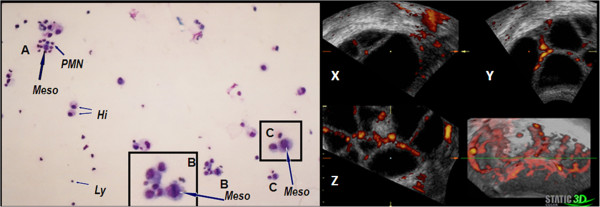
**Cytologic examination of cells in the ascitic fluid of a patient with ovarian hyperstimulation and ultrasonographic assessment of OHSS.** On the left side (A,C) atypical cells can be observed among the leukocytes (Ly), red blood cells, mesothelial cells (Meso) and histiocytes (Hi). Abnormal cells contain a dark cytoplasm that, in some places, resembles a seal-ring as well as large and rough-grained nuclei with multiple nucleoli. Mitosis and polymorphonuclear (PMN) epithelial-like cells can be seen in the smear (B; magnification 200X). The cytologic diagnosis was P IV: strong suspicion of an epithelial-like, malignant ovarian tumour. On the right side, three-dimensional (X,Y,Z; three orthogonal planes) Power Doppler ultrasonographic visualization of the ovaries in a case of severe OHSS that demonstrates ominous signs of hypervascularization (static 3D image).

Nine of the 23 patients who developed OHSS underwent culdocentesis for severe OHSS. These patients ranged in age from 23 to 34 years old (mean, 27.1 years). Ascitic fluid was obtained from these patients for further analysis. The cytological findings for these patients suggested the presence of ovarian malignancy (Table 1) with cytologic grades of P III and P IV in four cases each and intermediate findings (P III - P IV) in one case (Figure 1). None of the nine patients had ultrasonographic evidence of a possible ovarian tumour before starting treatment; therefore, we did not perform immediate surgical intervention or histological sampling and elected to follow the patients with supportive therapy.

**Table 1:** Cytological, histological, and follow-up results, including the serum levels of ovarian cancer tumour markers and the ROMA index (%), for women with ovarian hyperstimulation syndrome

**Case no.**	**Age (y)**	**Douglas puncture**	**OIT**	Ascites cytology*	**Control histology**	**Remarks**	**CA-125 (U/mL)**	**HE4 (pM)**	**ROMA (%)**
1	28	05/2006	CC + hMG + hCG	P IV	06/2006, neg.^#^	Tumor-free	45.2	40.1	4.9
2	23	03/2007	FSH + hMG + hCG	P IV	05/2007, neg.^#^	Tumor-free	27.1	47.9	7.0
3	24	10/2007	CC + FSH	P IV	12/2007, neg.^#^	Tumor-free	505.8	38.2	5.0
4	23	10/2007	CC + hCG	P IV	12/2007, neg.^#^	Tumor-free	40.5	39.9	4.8
5	26	11/2007	CC + FSH + hCG	P III	02/2008, neg.^#^	Tumor-free^§^	19.2	51.6	8.1
6	30	02/2008	hMG + hCG	P III	04/2008, neg.^#^	Tumor-free	9.8	45.6	5.2
7	26	11/2008	FSH + hCG	P III	01/2009, neg.^#^	Tumor-free	38.1	45.2	6.2
8	30	11/2009	GnRH-a + hMG + hCG	P III	01/2010, neg.^#^	Tumor-free	56.3	37.4	4.2
9	34	10/2012	GnRH-a + FSH + hCG	P III-IV	12/2012, neg.^#^	Tumor-free^$^	210.3	40.1	5.3

The dates of the Douglas puncture and control histology tests are shown.

*OIT* Ovarian induction therapy, *CC* Clomiphene citrate, *hMG* Human menopausal gonadotropin, *hCG* Human chorionic gonadotropin, *GnRH-a* Gonadotropin-releasing hormone analogue, *FSH* Follicle-stimulating hormone.

*See Methods for a description of the cytologic stages P I-P V.

^#^Negative histology, normal ovarian tissue.

^§^Trigeminal pregnancy.

^$^Missed abortion.

The reference value for CA-125 is 0–39 U/ml and for HE4 is 0–150 pM.

Figure 1 shows a photograph from a representative aspiration from case no. 2, a 23-year-old woman who underwent OIT in preparation for homologous insemination. Cells sampled from the ascitic fluid were graded as P IV. In all cases, the volume of the ovarian ascitic fluid gradually decreased. To ensure that no malignancy existed, a laparoscopic examination was performed at 8 to 12 weeks. After close inspection of the abdominal cavity, eluents from the Douglas pouch were sampled and histological samples were obtained from the ovaries. All cytological and histological tests of these follow-up examinations were benign (Table 1). Follow-up laparoscopies were not performed for these three patients, who were instead followed clinically. During the follow-up period, none of the nine patients displayed signs of an ovarian malignancy.

Peripheral blood serum levels of CA-125 and HE4 tumour markers were also evaluated for the nine patients with severe OHSS. The mean (± SD) value of CA-125 was increased (105.81 ± 161.55 U/mL) compared to the reference range of 0 to 39 U/mL. However, the mean serum level of HE4 (42.89 ± 4.88 pM) was within the normal range of 0 to 150 pM. The ROMA predictive index was determined based on the concentrations of these two tumour markers and the subject’s premenopausal status. The ROMA index was very low (5.63% ± 1.24%), which indicated that the patients were not at a high risk for developing ovarian malignancies (Table 1).

## Discussion

Our data over a 6-year period indicate that there is no relationship between OIT and subsequent malignant ovarian tumour development among patients at our centre. Although the cytologic results of the ascitic cells from patients with severe OHSS were initially suggestive of malignancy, these patients did not develop any evidence of malignant ovarian tumours.

Because the risk of malignant ovarian tumours in is higher in nulliparous women, greater vigilance is necessary when treating this group. Bimanual examination and vaginal ultrasonography are essential. The best method to examine for enlarged ovaries is colour-Doppler ultrasound. Rarely, laparoscopy or laparotomy may be necessary. Therapy should only begin after malignant ovarian tumours have been ruled out and/or benign tumours have been removed.

Tumour markers, although useful, cannot differentiate ovarian enlargement caused by overstimulation from enlargement due to a malignancy. For example, the serum CA-125 level closely correlates with the volume of the ovary and is not indicative of the underlying pathology [19]. Previous studies found no statistically significant differences when comparing serum CA-125 levels between spontaneous and stimulated cycles or between pregnant and non-pregnant patients [20]. Longitudinal follow-up of patients with sequential determinations of their tumour markers may be helpful for an accurate assessment. During the follow-up of patients with hyper-stimulated ovaries, the serum concentration of the CA-125 tumour marker declines and eventually normalizes. In patients with malignant disease, the serum levels remain elevated or gradually increase.

To overcome the relatively low specificity and sensitivity of risk assessment by a single tumour marker, Moore *et al.* introduced the ROMA index as an accurate predictive index for ovarian cancer (76.4% sensitivity and 96% specificity) [18]. Our results confirmed that the single biomarker determination of CA-125 was not sufficient to reliable evaluate ovarian malignancy in OHSS. When the combination of CA-125 and HE4 was used, despite the high levels of CA-125, HE4 remained under the reference value and indicated no obvious signs of malignancy. This observation was demonstrated by the low ROMA scores (Table 1).

Epidemiologic follow-up data of infertile patients demonstrates an increased life-long risk for high-grade or borderline malignant ovarian malignancies. However, the exact reason for this increased risk is unclear. Whereas some authors believe it is a result of the infertility itself [21], others suggest that ovulation induction is associated with cancerogenesis [22,23]. Although patients undergoing OIT may be at an increased risk of developing ovarian tumours, studies have shown reassuring results in terms of hormone treatment and the incidence of invasive epithelial ovarian cancer [24]. However, exogenous hormone treatment is associated with an increased risk of borderline ovarian malignancy [25]. OIT has not been shown to increase the risk of breast, uterine, or invasive ovarian cancers, although the risk of borderline ovarian tumours might increase [26]. Moreover, the risk of cancer has been shown to be similar in children conceived by artificial reproductive therapies and those conceived naturally [27]. It should also be noted that, due to close medical surveillance, malignancies are overdiagnosed in the female population; this may also augment the early detection of cancers [28].

Our data suggest that even when the cytological evaluation of ascitic cells obtained in patients diagnosed with OHSS indicates abnormality and possible malignancy, radical surgical intervention is not clinically indicated. Instead, these patients should be closely followed and monitored. If the ovarian size remains abnormal, then the aetiology of the enlargement should be determined by histological sampling via laparoscopy, and the histologist should be informed of the previous OIT. Surgery may still be required for abdominal bleeding, ovarian torsion or rupture, or extra-uterine pregnancy.

## Conclusions

We observed a minimal risk of ovarian malignancy shortly after OIT at our IVF clinic. Large population-based studies will be required to determine if ovarian induction is associated with tumourigenesis over the long-term.

## Competing interests

The authors declare that they have no competing interests.

## Authors’ contributions

IH has made substantial contributions to the conception and the design of this study. EK carried out the histopathologic analysis of the ascitic samples along with KC, who also participated in the acquisition of data and the pathologic assessments. KK provided the clinical data of the patients who participated in the assisted reproduction program and helped in the statistical analysis. BF participated in the design of the study and carried out the immunoassays; in addition, he has also been involved in drafting the manuscript. PMG conceived the study, participated in its design and coordination, and helped to draft the manuscript. All authors read and approved the final manuscript and have given final approval of the version to be published.
